# Electromyographic analysis of the serratus anterior and upper trapezius in closed kinetic chain exercises performed on different unstable support surfaces: a systematic review and meta-analysis

**DOI:** 10.7717/peerj.13589

**Published:** 2022-06-30

**Authors:** Guillermo Mendez-Rebolledo, Ignacio Orozco-Chavez, Juan Morales-Verdugo, Rodrigo Ramirez-Campillo, Ann M.J. Cools

**Affiliations:** 1 Escuela de Kinesiología, Facultad de Salud, Universidad Santo Tomás, Chile; 2 Departamento de Ciencias del Movimiento Humano, Facultad de Ciencias de la Salud, Universidad de Talca, Talca, Chile; 3 Departamento de Ciencias Preclínicas, Facultad de Medicina, Universidad Católica del Maule, Talca, Chile; 4 Exercise and Rehabilitation Sciences Laboratory, School of Physical Therapy, Faculty of Rehabilitation Sciences, Universidad Andres Bello, Santiago, Chile; 5 Department of Rehabilitation Sciences, Faculty of Medicine and Health Sciences, Ghent University, Ghent, Belgium

**Keywords:** Shoulder, Scapula, Rehabilitation, Physical therapy modalities, Resistance training, Musculoskeletal and neural physiological phenomena

## Abstract

**Background:**

Multiple investigations have compared the electromyographic (EMG) activity of the scapular muscles between stable and unstable support surfaces during the execution of closed kinetic chain exercises. However, these comparative analyses have grouped different unstable surfaces (wobble board, BOSU, therapeutic ball, and suspension equipment) into a single data pool, without considering the possible differences in neuromuscular demand induced by each unstable support surface. This study aimed to analyze the individual effect of different unstable support surfaces compared to a stable support surface on scapular muscles EMG activity during the execution of closed kinetic chain exercises.

**Methodology:**

A literature search was conducted of the Pubmed Central, ScienceDirect and SPORTDiscus databases. Studies which investigated scapular muscles EMG during push-ups and compared at least two support surfaces were included. The risk of bias of included articles was assessed using a standardized quality assessment form for descriptive, observational and EMG studies, and the certainty of the evidence was measured with the Grading of Recommendations Assessment, Development and Evaluation (GRADE) approach. A random-effects model was used to calculate effect sizes (ES, Hedge’s *g*).

**Results:**

Thirty studies were selected in the systematic review. Of these, twenty-three low-to-high quality studies (498 participants) were included in the meta-analysis. The main analyzes revealed, in decreasing order, greater UT EMG activity during push-ups performed on suspension equipment (ES = 2.92; *p* = 0.004), therapeutic ball (ES = 1.03; *p* < 0.001) and wobble board (ES = 0.33; *p* = 0.003); without effect on the BOSU ball. In addition, no effect was observed for SA on any unstable device. The certainty of the evidence ranged from low to very low due to the inclusion of descriptive studies, as well as high imprecision, inconsistency, and risk of publication bias.

**Conclusion:**

These findings could be applied in scapular muscles strengthening in healthy individuals. The use of suspension equipment achieves higher UT activation levels. Conversely, the use of any type of unstable devices to increase the activation levels of the SA in shoulder musculoskeletal dysfunctions is not recommended. These conclusions should be interpreted with caution as the available evidence showed a low to very low certainty of evidence, downgraded mostly by inconsistency and imprecision.

## Introduction

Scapular muscle imbalance is a frequently reported alteration in individuals with shoulder pain, overhead athletes, and physically active and healthy populations (Ludewig & Cook, 2000; Cools et al., 2007; Cools et al., 2014; De Mey et al., 2013; Kinsella & Pizzari, 2017). A correct muscle balance between the scapular muscles (*e.g.*, upper trapezius (UT), lower trapezius (LT), and serratus anterior (SA)) is important for normal shoulder function (Ludewig & Cook, 2000; Cools et al., 2007). For instance, a decrease in the activation of the SA or LT, and the consequent overactivation of the UT, generates an alteration of the position and scapular movement, *i.e.*, dyskinesia, characterized by excessive upward rotation and anterior scapular tilt during arm elevation which can lead to subacromial impingement or glenohumeral instability (Ludewig & Cook, 2000; Kibler et al., 2013; Kinsella & Pizzari, 2017; Huang, Ou & Lin, 2019).

It is widely recognized that scapular dyskinesis rehabilitation programs focused on decreasing UT activation and increasing SA and LT activation in early stages of the rehabilitation process contribute to restoring normal scapular position and movement (Cools et al., 2007; Youdas et al., 2020b; Berckmans et al., 2021). Several closed kinetic chain exercises have been reported as efficient to increase SA and LT myoelectrical activity, such as “push-up” (Calatayud et al., 2014a; Santos et al., 2018), “push-up plus” (scapular protraction posture in the end of the ascending phase of a push-up) (Cools et al., 2014; Batbayar et al., 2015; Gioftsos et al., 2016; Hwang et al., 2017), “scapular protraction” (scapular protraction and retraction with elbows extended) (Andersen et al., 2012; Lee, Lee & Park, 2013; De Mey et al., 2014), and “plank” (prone position, with the trunk and leg fully extended, the shoulders flexed at 90° and elbows in extension) (Pontillo et al., 2007; Tucker et al., 2010; Ashnagar et al., 2016; Oliver et al., 2018).

As a mean of progression in UT and SA strengthening and rehabilitation programs, unstable support surfaces have been incorporated into the push-up variants. Compared to stable surface, increased SA and UT EMG during push-ups has been reported when performed on unstable surfaces such as both-sides-up (BOSU) ball (Tucker et al., 2010; Borreani et al., 2015a), wobble board (Park & Yoo, 2011; Biscarini, Contemori & Grolla, 2019), therapeutic ball (Seo et al., 2013), and suspension equipment (Jeong, Chung & Shim, 2014; De Mey et al., 2014). However, contradictory results have been reported, observing a significant decrease or no difference in the EMG of the SA when comparing different types of unstable surfaces against stable surface (Pirauá et al., 2014; Gioftsos et al., 2016; Horsak et al., 2017).

In sport rehabilitation, devices with a greater base of support (*e.g.*, BOSU ball) are used as a progression to stable surfaces in earlier stages of rehabilitation or training, while those with a smaller base (*e.g.*, suspension equipment) or greater mobility (*e.g.*, therapeutic ball) are integrated in more advanced stages (Behm et al., 2010). In this context, there is evidence that suspension mechanisms and the therapeutic ball could generate a greater neuromuscular recruitment demand compared to other support surfaces such as BOSU or floor (Lehman, Hoda & Oliver, 2005; Imai et al., 2010; Behm & Colado, 2012; Czaprowski et al., 2014; Borreani et al., 2015a; Youdas et al., 2020b). However, the comparative analyzes observed in previous reviews (Kang et al., 2019; Cappato de Araújo et al., 2021) have grouped in a single data pool different types of unstable support surfaces—BOSU, therapeutic ball, suspension equipment, among others—without considering the possible and potential differences in neuromuscular demand induced by the individual analysis of each unstable support surface (Mendez-Rebolledo et al., 2021). In this context, to our knowledge, there is no quantitative analysis of the evidence that groups the data according to these differences. In summary, previous reviews chose to analyze EMG activity in subgroups that considered the type of exercise (push-up, push-up plus, knee push-up, among others), type of execution (isometric and dynamic), and type of surface (stable and unstable). However, the influence each type of unstable surface (BOSU, wobble board, therapeutic ball, or suspension equipment) on the EMG activity still unclear. Therefore, the purpose of this systematic review with meta-analysis was to analyze the individual effect of different unstable support surfaces (*i.e.*, BOSU ball, wobble-board, therapeutic ball, and suspension equipment) compared to a stable support surface on scapular muscles EMG activity during the execution of closed kinetic chain exercises.

## Survey methodology

### Study design

**Figure 1:**
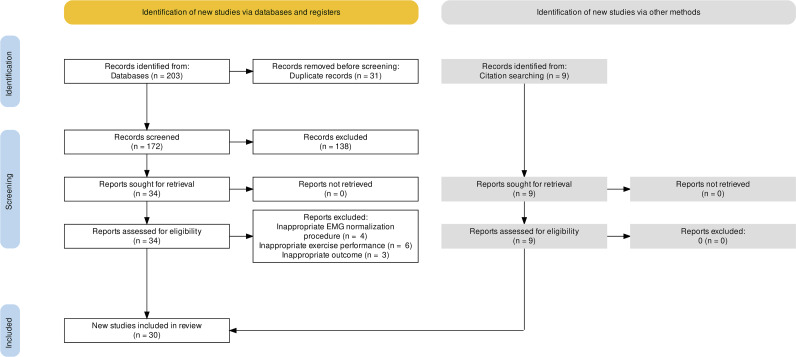
PRISMA flow diagram.

This systematic review and meta-analysis followed the Preferred Reporting Items for Systematic Reviews and Meta-Analyses (PRISMA) updated to year 2020 (Page et al., 2021). The whole process of study selections was summarized in the PRISMA flow diagram (Fig. 1).

### Inclusion and exclusion criteria

This review includes studies in English applying the PICOS (Participants; Intervention/exposure; Comparator; Outcome; Study design) approach. The studies were included according to the following inclusion criteria; (i) population: healthy volunteers between 18 and 55 years old to reduce the possible effects of shoulder pathologies associated with aging. No restrictions of sex, ethnicity or socioeconomic status were applied; (ii) intervention/exposure: closed kinetic chain exercise performance on unstable support surfaces for upper limb. The unstable support surfaces were categorized as BOSU ball, wobble board, therapeutic ball and suspension equipment based in previous investigations (Borreani et al., 2015a; Horsak et al., 2017; Youdas et al., 2020a); (iii) comparators: the same exercise performed on a stable support surface for upper limb; (iv) outcomes: normalized EMG amplitude of scapular muscles (UT, LT, middle trapezius (MT), and SA) based on maximum voluntary isometric contraction (MVIC), as this is the gold standard method of measuring myoelectrical activity; and (v) study design: descriptive studies (cross-sectional), observational studies (case and control), and experimental studies. The following exclusion criteria was applied; (i) participants: individuals with a clinical condition that could interfere in the execution of the exercise (*e.g.*, shoulder pain or scapular dyskinesis) without control group; (ii) intervention/exposure: use of more than one unstable support surface for each instability condition; (iii) comparators: use of unstable support surface for lower limb or another body segment; (iv) outcomes: lack of description of EMG normalization in the procedures of the selected studies or normalization based on reference voluntary isometric contraction.

### Information sources and search strategy

To identify relevant studies, a first search was carried out from November 2020 to February 2021 in the following electronic databases: Pubmed Central, ScienceDirect and SPORTDiscus, considering articles from January 1995 to September 2021. The same search was updated from 1 to 31 October 2021. The search strategy was carried out according to the terms: scapular muscles, EMG activity, exercises, and unstable surface. See Appendix A for the detailed search strategy in each database.

### Selection process

Two researchers (OC and MV) independently reviewed titles and abstracts of all articles retrieved. Then, they independently screened full-text articles for inclusion. In case of disagreement, consensus on which articles to include was reached by discussion. If necessary, a third researcher (MR) was consulted to make the final decision.

### Data collection process

An author (OC) completed the extraction of data from selected studies. Due to the heterogeneity of the nomenclature and design of the exercises reported in the literature, these were categorized according to previously described criteria (Mendez-Rebolledo et al., 2021; Cappato de Araújo et al., 2021). A second author (MV) checked the accuracy and consistency of all entries and made relevant clarifications when necessary. When data were displayed in a figure and no numerical data were provided by authors after being contacted, a validated (*r* = 0.99, *p* < 0.001) software (WebPlotDigitizer; https://apps.automeris.io/wpd/) was used to derive numerical data from figures (Drevon, Fursa & Malcolm, 2017).

### Data items

This review only included one eligible outcome, which corresponded to neuromuscular activity (normalized EMG activity of scapular muscles). In case of multiple outcomes (experimental studies), baseline EMG activity was collected. Additionally, the following data were extracted in an ad-hoc table (Table 1): The study (author, year); Participant’s characteristics (age, height, weight); Performed exercises (*i.e.*, push up, plank); Support surface (support device); and Scapular muscles evaluated (UT, MT, LT, and SA).

**Table 1:** Summary of the characteristics and results of the selected studies.

**Author (year)**	**Participants** **(Age; height; weight)**	**Exercises**	**Support surface (support device)**	**Scapular muscles evaluated**	**Results**
Biscarini, Contemori & Grolla (2019)	*n* = 18 (11 M, 7 F; 21-52 y; 159–187 cm; 51-86 kg)	Plank	Stable (Floor)	MT, SA	The use of unstable surfaces significantly increased the EMG activity of SA muscle
			Unstable (Wobble board)		
Borreani et al. (2015a); Borreani et al. (2015b)	*n* = 29 M (23.5 ± 3.1 y; 178.2 ± 5.9 cm; 75.2 ± 8.5 kg)	Push-up	Stable (Floor)	UT	The use of unstable surfaces significantly increased the EMG activity of UT muscle
		Half push-up	Unstable (Suspension)		
Borreani et al. (2015a); Borreani et al. (2015b)	*n* = 30 M (23 ± 1.13 y; 178.87 ± 8.21 cm; 78.01 ± 8.5 kg)	Push-up	Stable (Floor)	SA	The use of unstable surfaces such as stability disc, fitness dome, and wobble board significantly increased the EMG activity of the SA.
			Unstable (Stability Disc; Wobble Board; BOSU ball; Suspension equipment)		Suspension only increased trunk EMG activity
Byrne et al. (2014)	*n* = 21 (10 M, 11 F; 21.9 ± 2.4 y; 175.5 ± 10.13 cm; 74.2 ± 12.61 kg)	Plank	Stable (Floor)	SA	There were no statistically significant differences between stable and unstable surface.
			Unstable (Suspension equipment)		Post hoc analysis revealed that foot suspension generated greater SA EMG activation than arm suspension.
Calatayud et al. (2014a); Calatayud et al. (2014b)	*n* = 29 M (22.6 ± 2.6 y; 176.0 ± 4.4 cm; 74.6 ± 6.7 kg)	Push-up	Stable (Floor)	UT, SA	There were no differences in UT EMG activity between stable and unstable surfaces.
		Half Push-up	Unstable (Suspension equipment)		The use of unstable surfaces decreased the EMG activity of SA muscle
Calatayud et al. (2014a); Calatayud et al. (2014b)	*n* = 29 M (23.5 ± 3.1 y; 178.2 ± 5.9 cm; 75.2 ± 8.5 kg)	Push-up	Stable (Floor)	UT	The use of unstable surfaces significantly increased the EMG activity of UT
			Unstable (Suspension equipment)		
De Araújo et al. (2011)	*n* = 20 M (22 ± 3 y; 175 ± 5 cm; 68 ± 7 kg)	One arm isometric	Stable (Floor)	UT, SA	There were no statistically significant differences between the stable and unstable surface for any of the evaluated muscles
			Unstable (Therapeutic Ball)		
De Araújo et al. (2018)	*n* = 18 M (21.50 ± 2.65 years; 173 ± 3 cm; 74.9 ± 2.69 kg)	Push-up	Stable (Floor)	UT, LT, SA	The use of unstable surfaces significantly increased the EMG activity of UT and SA muscles.
			Unstable (Wobble board)		
De Araújo et al. (2020)	*n* = 23 M (21.74 ± 3 y; 175 ± 6 cm; 71.20 ± 7.79 kg)	Push-up plus	Stable (Floor)	UT, LT, SA	The use of unstable surfaces (BOSU ball) increased the EMG activity of SA. No significant differences were observed for the UT muscle.
			Unstable (BOSU ball)		
De Mey et al. (2014)	*n* = 47 (26 M, 21 F; 22 ± 4.31 y; 176 ± 8.3 cm; 69 ± 8.57 kg)	Push-up	Stable (Bar)	UT, LT, SA	The use of unstable surfaces increased the EMG activity of UT in Push-up. No significant differences were observed for the SA muscle.
		Scap protraction	Unstable (Suspension equipment)		The use of unstable surfaces decreased the EMG activity of SA in scap protraction.
De Faria et al. (2021)	*n* = 14 M (24.57 ± 4.30 y; 176 ± 6 cm; 82.79 ± 9.04 kg)	Push-up	Stable (Floor)	UT, LT, SA	There were no statistically significant differences between the stable and unstable surface for any of the evaluated muscles.
			Unstable (Wobble Board)		
Gioftsos et al. (2016)	*n* = 13 M (20.5 ± 1.0 y; 178.8 ± 7.2 cm; 79.2 ± 12.3 kg)	Push-up	Stable (Floor)	UT, LT, SA	There were no statistically significant differences between the stable and unstable surface for any of the evaluated muscles.
						Scap protraction	Unstable (Wobble Board)		
		Push-up plus			
Herrington, Waterman & Smith (2015)	*n* = 21 (10 M, 11 F; 22.8 ± 1.4 y)	Plank	Stable (Floor)	SA	The use of the foam surface decreased the EMG activity of SA during the one hand isometric exercise.
		One arm isometric	Unstable (Therapeutic Ball, Foam)		No significant differences were observed in the EMG activity of the SA muscle on unstable surfaces during a plank exercise.
Horsak et al. (2017)	*n* = 19 F (23 ± 3 y; 167 ± 6 cm; 60 ± 6 kg)	Scap protraction	Stable (Floor)	UT, LT, SA	There were no statistically significant differences in periscapular EMG activity when comparing between stable and unstable support surface
		Push-up plus	Unstable (Suspension equipment)		
Karagiannakis, Athanasopoulos & Mandalidis (2018)	*n* = 15 F (24.0 ± 5.2 y; 172.5 ± 5.5 cm; 65.6 ± 5.1)	Push-up	Stable (Floor)	UT, SA	No interaction was observed between group, dominance, or type of surface. Periscapular EMG activity was not influenced by unstable surfaces
			Unstable (BOSU ball)		
Kim et al. (2014)	*n* = 15 M (23.27 ± 1.28 y; 174.27 ± 3.51 cm; 67.33 ± 4.76 kg)	Knee Push-up plus	Stable (Floor)	SA	There were no statistically significant differences in SA EMG activity between stable and unstable surface.
			Unstable (BOSU ball)		
Kim & Yoo (2019)	*n* = 11 M (22 ± 1.9 y; 174.57 ± 4.32 cm; 62.2 ± 4.7 kg)	Push-up	Stable (Floor)	LT	There were no statistically significant differences in LT EMG activity between stable and unstable surface.
		Scap protraction	Unstable (Wobble Board)		
Lee, Lee & Park (2013)	*Unstable group:n* = 10 M (23.7 ± 1.21 y; 175.16 ± 4.42 cm, 73.01 ± 8.67 kg)	Push-up plus	Stable (Floor)	UT, LT, SA	The use of unstable surfaces increased the EMG activity of the SA muscle.
	*Stable group:n* = 10 M (23.3 ± 1.45 y, 174.27 ± 3.29 cm, 74.41 ± 7.49 kg)		Unstable (Suspension equipment)		There were no statistically significant differences in EMG activity of UT and LT between stable and unstable surface.
Lehman, Gilas & Patel (2008)	*n* = 10 M (26.3 ± 1.1 y; 83.3 ± 10.9; 174.7 ± 12.9 cm)	Push-up	Stable (Floor)	UT, LT, SA	There were no statistically significant differences between stable and unstable support surface for any of the scapular muscles evaluated during push-up and scap protraction exercises
		Scap protraction	Unstable (Therapeutic ball)		
Maenhout et al. (2010)	*n* = 32 (16 M, 16 F; 22,88 ± 2,43 y; 173 ± 9 cm; 65,59 ± 8,14 kg)	Knee Push-up plus	Stable (Floor)	UT, LT, SA	The use of unstable surface (wobble board) decreased the EMG activity of SA.
			Unstable (Wobble board)		
Martins et al. (2008)	*n* = 12 M (175 ± 54 cm; 22.8 ± 3.1 y; 68.7 ± 7.9 kg)	One arm isometric	Stable (Floor)	UT, SA	No significant differences were observed in the EMG activity of UT or SA when using unstable support surfaces.
			Unstable (Therapeutic ball)		
Park & Yoo (2013)	*n* = 16 M (26 y; 176.1 ± 5.4 cm; 64.6 ± 4.9 kg)	Push-up	Stable (Floor)	UT, LT, SA	There was an increase in muscle activity of all scapular muscles when using the unstable support surface.
			Unstable (Wobble Board)		
Park & Yoo (2013)	*n* = 14 M (22 ± 2 y; 174.6 ± 57 cm; 62.2 ± 4.8 kg)	Push-up	Stable (Floor)	UT, SA	The use of unstable surface (wobble board) increased the EMG activity of UT and SA.
			Unstable (Wobble board)		
Patselas et al. (2021)	*n* = 13 M (21.1 ± 1.8 y; 180 ± 4 cm; 79 ± 12kg)	Push-up	Stable (Floor)	UT, SA	There were no statistically significant differences between stable and unstable support surface for any of the scapular muscles evaluated.
		Push-up plus	Unstable (Wobble board)		
Sandhu, Mahajan & Shenoy (2008)	*n* = 35 M (20-30 y; 173.65 ± 256 cm; 69.9 ± 0.2 kg)	Push-up	Stable (Floor)	UT, SA	There were no statistically significant differences between stable and unstable support surface for any of the scapular muscles evaluated.
		Knee Push-up	Unstable (Therapeutic ball)		
		Plank			
Seo et al. (2013)	*n* = 10 M (24.6 y; 176.2 ± 3.67 cm; 75.7 ± 5.16 kg)	Half Push-up	Stable (Chair)	UT, MT, LT, SA	The use of unstable surface (therapeutic ball) increased the EMG activity of UT, MT, LT and SA during half and knee push-up performance.
		Knee Push-up	Unstable (Therapeutic ball)		
Pirauá et al. (2014)	*n* = 30 M (21.7 ± 2.5 y; 70.5 ± 9 kg; 173 ± 1 cm)	Push-up	Stable (Floor)	UT, LT, SA	The use of unstable surface (wobble board) increased the EMG activity of UT and LT and decreases the EMG activity of SA.
			Unstable (Wobble board)		
Tucker et al. (2010)	*Healthy Group*: *n* = 15 (11 M, 4 F; 21.0 ± 2.5 y; 176.0 ± 7.8 cm; 76.1 ± 13.4 15 kg)	Push-up	Stable (Floor)	UT, MT, LT, SA	The use of BOSU ball increased the EMG activity of UT, MT and LT muscles and decreased the EMG activity of SA.
	*Impingement Group*: *n* = 15 (11 M-4 F; 20.4 ± 3.8 y; 174.1 ± 9.7 cm; 73.3 ± 11.7 kg)		Unstable (BOSU ball, Cufflink)		The use of cufflink decreased the EMG activity of the UT, MT, LT muscle and increased the EMG activity of SA.
Youdas et al. (2020a); Youdas et al. (2020b)	*n* = 22 M (24.6 ± 3.2 y, 180 ± 10 cm; 87.9 ± 9.3 kg)	Push-up	Stable (Floor)	SA	SA recruitment decreased during a push-up with performance on suspension equipment and dual instability devices compared to the standard push-up.
	*n* = 10 F (23.6 ± 1.4 y; 160 ± 10 cm; 60 ± 4.2 kg)		Unstable (BOSU ball, Suspension equipment)		
Youdas et al. (2018)	*n* = 13 M (25.4 ± 5.7 y; 190 ± 10 cm; 89.6 ± 6 kg)	Plank	Stable (Floor)	SA	A high activation of SA was observed during the prone plank on floor and on therapeutic ball. There were no statistically significant differences between both conditions.
	*n* = 13 F (25 ± 3.8 y; 170 ± 10 cm; 63.5 ± 7.3 kg)		Unstable (Therapeutic ball)		

**Notes.**

M male F female y years UT upper trapezius muscle MT middle trapezius muscle LT lower trapezius muscle SA serratus anterior muscle

### Study risk of bias assessment

Two independent authors (OC and MV) assessed the risk of bias of all included papers using a standardized quality assessment form for observational and descriptive studies (Siegfried et al., 2005) and it was adapted specifically for this study following recommendations of previous reports regarding risk of bias assessment of EMG studies (Table 2) (Ganderton & Pizzari, 2013; Edwards et al., 2017; Karabay, Emük & Özer Kaya, 2020; Cappato de Araújo et al., 2021). The Non-Randomized Studies Methods Group of The Cochrane Collaboration has commended the quality assessment tool (Reeves et al., 2021). This tool was chosen as it evaluates external validity, performance bias and detection bias. Furthermore, it itemizes and displays each aspect of risk of bias in its raw form for readers. Risk of bias classification was based on the sum of the scores (0 = criterion not observed; 1 = criterion observed). Studies with score from 0–5, 6–9, and 10–12 were classified as high, moderate, and low risk of bias, respectively. Score disagreements were resolved by consensus, and the final agreed-upon rating was assigned to each study.

**Table 2:** Standardized quality assessment form for observational studies.

**Study**	**External validity**		**Internal validity**										
			**Performance**	**Detection**		**Selectin bias/control of confounding**							
	**Representative Sample**	**Participation rate**	**Direct observation**	**Blind** **assessors**	**Physical examination for participation**	**Randomization of exercise**	**Familiarization of exercises**	**Standardization of exercise technique**	**Randomization of MVIC**	**Appropriate normalization procedure**	**Appropriate statistical tests**	**Trial to trial reliability**	**Total (maximum = 12)**
Biscarini, Contemori & Grolla (2019)	1	1	1	0	0	1	1	0	0	1	1	0	**7**
Borreani et al. (2015a); Borreani et al. (2015b)	0	1	1	0	0	1	1	1	0	1	1	0	**7**
Borreani et al. (2015a); Borreani et al. (2015b)	0	1	1	0	0	1	1	1	0	1	1	0	**7**
Byrne et al. (2014)	1	0	1	0	0	1	1	1	0	1	1	0	**7**
Calatayud et al. (2014a); Calatayud et al. (2014b)	0	1	1	0	0	1	1	1	0	1	1	0	**7**
Calatayud et al. (2014a); Calatayud et al. (2014b)	0	1	1	0	0	1	1	1	0	1	1	0	**7**
De Araújo et al. (2011)	0	1	1	0	1	1	0	1	0	1	1	0	**7**
De Araújo et al. (2018)	0	1	1	1	1	1	0	0	1	1	1	0	**8**
De Araújo et al. (2020)	0	1	1	0	1	1	1	1	1	1	1	0	**9**
De Mey et al. (2014)	1	1	1	0	0	0	0	1	0	1	1	0	**7**
De Faria et al. (2021)	0	1	1	0	1	1	0	1	1	1	1	0	**8**
Gioftsos et al. (2016)	0	1	1	0	0	1	1	1	0	1	1	1	**8**
Herrington, Waterman & Smith (2015)	1	1	1	0	0	0	0	1	0	1	1	0	**6**
Horsak et al. (2017)	0	1	1	0	0	1	1	1	1	1	1	1	**9**
Karagiannakis, Athanasopoulos & Mandalidis (2018)	0	1	1	0	1	1	1	1	0	1	1	0	**8**
Kim et al. (2014)	0	1	1	0	1	1	1	1	1	1	1	0	**9**
Kim & Yoo (2019)	0	1	1	0	0	1	0	1	1	1	1	0	**7**
Lee, Lee & Park (2013)	0	1	1	0	0	1	1	0	0	1	1	0	**6**
Lehman, Gilas & Patel (2008)	0	1	1	0	0	0	0	1	0	1	1	0	**5**
Maenhout et al. (2010)	1	1	1	0	0	1	0	1	0	1	1	0	**7**
Martins et al. (2008)	0	1	1	0	1	1	1	1	0	1	1	1	**9**
Park & Yoo (2013)	0	1	1	0	1	1	0	1	0	1	1	0	**7**
Park & Yoo (2013)	0	1	1	0	0	1	1	1	0	1	0	0	**6**
Patselas et al. (2021)	0	0	1	0	0	1	1	1	0	1	1	1	**7**
Sandhu, Mahajan & Shenoy (2008)	0	1	1	0	0	1	1	1	0	1	1	0	**7**
Seo et al. (2013)	0	1	1	0	0	0	1	1	0	1	1	0	**6**
Pirauá et al. (2014)	0	1	1	1	1	1	1	1	1	1	1	0	**10**
Tucker et al. (2010)	1	1	1	0	1	1	1	1	0	1	1	0	**9**
Youdas et al. (2020a); Youdas et al. (2020b)	1	1	1	0	0	1	0	1	0	1	1	0	**7**
Youdas et al. (2018)	1	1	1	0	0	1	1	1	0	1	1	0	**8**

**Notes.**

MVIC maximum voluntary isometric contraction

A study was representative if it included both female and male participants in the sample. Physical examination indicates whether the participants were examined searching for any clinical condition. Randomization of exercises also considered support surface randomization. Standardization of the exercise technique indicates whether the cadence or velocity of execution of the exercise was determined. Appropriate normalization procedure indicates MVIC’s according to SENIAM recommendations.

### Certainty assessment

The certainty of the body of evidence was measured for two authors (OC and MV) with the Grading of Recommendations Assessment, Development and Evaluation (GRADE) approach for each meta-analysis performed (Guyatt et al., 2011; Zhang et al., 2019a; Zhang et al., 2019b), considering the levels “High”, “Moderate”, “Low” and “Very low”. As the included studies are descriptive, they were initially rated “Low”, and then can upgrade or downgrade according to the following criteria: if there is a large effect size, the certainty was upgraded by one level, as long as there are no downgrading criteria. The criteria for downgrading the confidence of evidence were: (i) risk of bias of included studies: one level of downgrade if the 25% or more of the included articles presented high risk of bias. That criteria was based in another study of shoulder muscle activity using EMG (Kamonseki et al., 2021a); (ii) inconsistency: one level of downgrade was applied if there was significant heterogeneity in the results, with *I*^2^ > 75%; (iii) indirectness: one level of downgrade was applied if there were differences between participants or outcomes of included studies; (iv) risk of publication bias: if there was conflict of interest, small studies sponsored, or if the *p* value of the Egger’s test was <0.05, one level of downgrade was applied (Ye et al., 2021); and (v) imprecision: one level of downgrade was considered if there was wide 95% confidence interval, that include both increase and decrease of muscle activity (Kamonseki et al., 2021b).

### Statistical analysis

Considering the particularities of reduced sample sizes in the field (Abt et al., 2020), a meta-analysis for a given muscle or surface being compared was conducted if at least three studies provided sufficient data for the calculation of Hedges’ *g* effect size (ES) (Moran, Ramirez-Campillo & Granacher, 2018; García-Hermoso, Ramírez-Campillo & Izquierdo, 2019). In this context, the EMG mean ± standard deviation (SD) of scapular muscles during push-ups on different surfaces were converted to ES. The data were standardized using post score SD. In all analyses, we used the random-effects model to account for differences between studies that might affect the effect (Deeks, Higgins & Altman, 2021). The ES values are presented alongside their respective 95% confidence intervals (CIs). Calculated ES were interpreted using the following scale: < 0.2, trivial; 0.2–0.6, small; > 0.6–1.2, moderate; > 1.2–2.0, large; > 2.0–4.0, very large; >4.0, extremely large (Hopkins et al., 2009). The heterogeneity was assessed using the *I*^2^ statistic, with values of <25%, 25–75%, and >75% considered to represent low, moderate, and high levels of heterogeneity, respectively (Higgins, 2003). Moreover, if there was a high level of heterogeneity (*i.e.*, *I*^2^ > 75%) a sensibility analysis was performed, with each study removed once from the meta-analysis to assess its impact on results heterogeneity. The risk of publication bias was explored using the extended (two-tailed) Egger’s test (Egger et al., 1997). To adjust for publication bias, a sensitivity analysis was conducted using the trim and fill method, with L0 as the default estimator for the number of missing studies (Duval & Tweedie, 2000). All analyses were carried out using the Comprehensive Meta-Analysis program (version 2; Biostat, Englewood, NJ, USA). The statistical significance threshold was set at *p* < 0.05.

## Results

### Study selection

The search strategy was completed on October 31 (2021) identifying 203 articles from databases. After removal of duplicate (*n* = 31), records were screened (*n* = 172), excluding 138 articles. The remaining 34 articles were retrieved and assessed for eligibility. Thirteen articles were excluded for inappropriate EMG normalization procedure (*n* = 4), inappropriate exercise performance (*n* = 6) and inappropriate outcome (*n* = 3). Nine records were identified from references citation, including a total of thirty articles meeting the inclusion criteria for the systematic review. The full search strategy and selection process are outlined in Fig. 1.

### Study characteristics

The total sample of this review was made up of 637 healthy individuals, with 127 females and 510 males, with an age range between 20 and 52 years. The closed kinetic chain exercises performed in the studies included in the systematic review were plank (*n* = 5), push-up (*n* = 18), half push-up (*n* = 3), knee push-up (*n* = 2), one arm isometric (*n* = 3), scap protraction (*n* = 5), push-up plus (*n* = 5) and knee push-up plus (*n* = 2). The support surfaces used in the execution of the exercises were: wobble board (*n* = 11), suspension equipment (*n* = 9), BOSU ball (*n* = 6), therapeutic ball (*n* = 7), stability disk (*n* = 1), foam (*n* = 1) and cufflink (*n* = 1) (Table 1). The scapular muscles analyzed in the selected studies included UT (*n* = 22), LT (*n* = 14), MT (*n* = 3), and SA (*n* = 27). In those studies that evaluated the upper and lower fibers of the SA, the upper part was considered since it is most evaluated in the included literature.

### Risk of bias in studies

The risk of bias scores of the included studies are shown in Table 2. Overall, the studies were of moderate risk of bias (mean ± SD = 7.4 ± 1.0 points), corresponding to 93.3% of all the studies submitted to the assessment scale. Only one study (Pirauá et al., 2014) presented a low risk of bias, and in the same way only one study (Lehman, Gilas & Patel, 2008) presented a high risk of bias. Despite this, the study was included in the quantitative analysis since there is no evidence that the unfulfilled criteria of the scale modify the results of the EMG activity. Regarding external validity, eight studies included a representative sample of men and women (Tucker et al., 2010; Maenhout et al., 2010; De Mey et al., 2014; Byrne et al., 2014; Herrington, Waterman & Smith, 2015; Youdas et al., 2018; Youdas et al., 2020a; Biscarini, Contemori & Grolla, 2019) and only two reported dropouts throughout the study (Byrne et al., 2014; Patselas et al., 2021). Regarding internal validity, ten of the included articles (Martins et al., 2008; Tucker et al., 2010; De Araújo et al., 2011; De Araújo et al., 2018; De Araújo et al., 2020; Park et al., 2013; Kim et al., 2014; Pirauá et al., 2014; Karagiannakis, Athanasopoulos & Mandalidis, 2018; De Faria et al., 2021) carried out a physical evaluation of the participants to identify any clinical condition. In addition, only three studies (Lee, Lee & Park, 2013; De Araújo et al., 2018; Biscarini, Contemori & Grolla, 2019) standardized movement velocity and phase duration of the exercises, and all of them included direct observation of participants during testing procedure. Ten articles did not report participant familiarization with exercise performance (De Araújo et al., 2011; De Araújo et al., 2018; De Faria et al., 2021; De Mey et al., 2014; Herrington, Waterman & Smith, 2015; Kim & Yoo, 2019; Lehman, Gilas & Patel, 2008; Maenhout et al., 2010; Park et al., 2013; Youdas et al., 2020a; Youdas et al., 2020b). Only four articles (Lehman, Gilas & Patel, 2008; Seo et al., 2013; De Mey et al., 2014; Herrington, Waterman & Smith, 2015) did not report the randomization of the exercises or support surface type. All the articles presented an appropriate normalization of the EMG signal, nevertheless only five studies (Pirauá et al., 2014; Horsak et al., 2017; De Araújo et al., 2018; De Araújo et al., 2020; De Faria et al., 2021) randomized MVIC measurement, and one study (Park et al., 2013) did not specify the statistical test performed for multifactorial analysis, only describing the pair comparison test. Lastly, four studies (Martins et al., 2008; Gioftsos et al., 2016; Horsak et al., 2017; Patselas et al., 2021) included trial to trial reliability analysis among EMG measurements.

### Quantitative synthesis: meta-analysis

For the meta-analysis, the articles were grouped considering the types of support surface [stable (floor) compared to wobble board, BOSU ball, therapeutic ball, or suspension equipment] for each scapular muscle (SA and UT) and push-up variants, since at least three studies provided sufficient data for the calculation of ES. Finally, twenty-three low-to-high quality studies (498 participants) were included in the meta-analysis.

#### Upper trapezius

##### Stable surface compared to suspension equipment.

**Figure 2:**
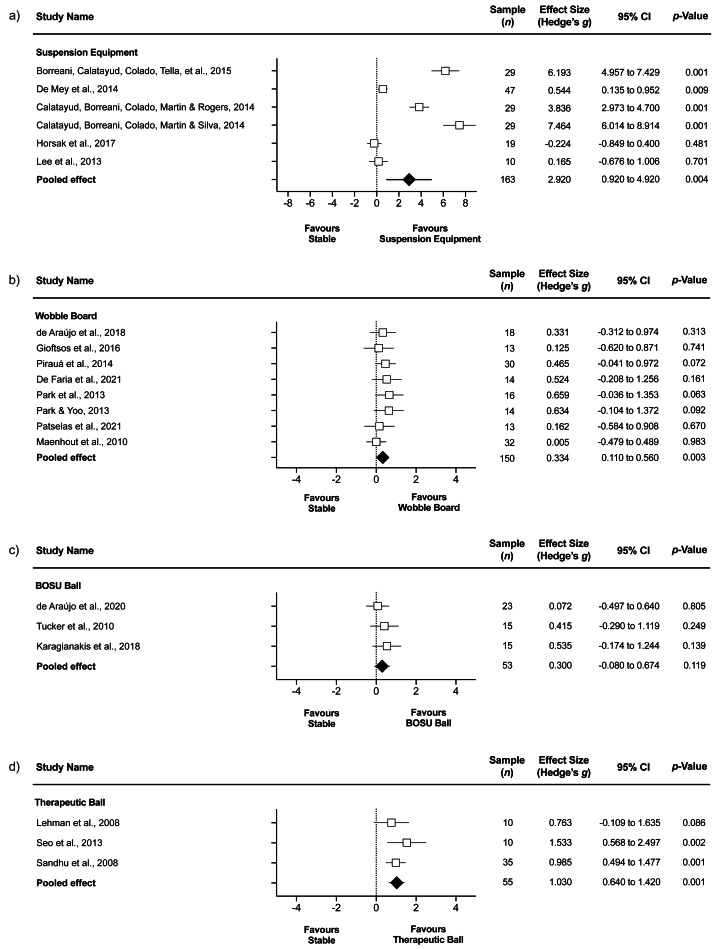
Upper trapezius EMG during push-ups performed on stable surface compared to (A) suspension equipment, (B) wobble board, (C) BOSU ball, and (D) therapeutic ball. Values shown are effect sizes (Hedges’s *g*) with 95% confidence intervals (CI). The size of the plotted squares reflects the statistical relative weight of the study. The green diamond reflects the overall result.

The comparison involved six studies (Lee, Lee & Park, 2013; Calatayud et al., 2014b; Calatayud et al., 2014a; De Mey et al., 2014; Borreani et al., 2015b; Horsak et al., 2017), involving 6 groups that completed push-ups on stable surface (*n* = 163), and 6 groups that completed push-ups on suspension equipment (*n* = 163). The forest plot (Fig. 2A) revealed greater UT EMG activity during suspension equipment compared to stable surface (ES = 2.92, very large; 95% CI [0.92–4.92]; *p* = 0.004; *I*^2^ = 97.6%; Egger’s test *p* = 0.055). After a sensibility analysis with each study removed once from the meta-analysis to assess its impact on results heterogeneity, the results remained similar, with *I*^2^ values > 90%. Moreover, a sensitivity analysis was conducted using the trim and fill method, and the results remained similar, with an ES = 2.06 and 95% CI [0.11–4.01].

##### Stable surface compared to wobble board.

The comparison involved eight studies (Maenhout et al., 2010; Park et al., 2013; Park & Yoo, 2013; Pirauá et al., 2014; Gioftsos et al., 2016; De Araújo et al., 2018; De Faria et al., 2021; Patselas et al., 2021), involving 8 groups that completed push-ups on stable surface (*n* = 150), and 8 groups that completed push-ups on wobble board (*n* = 150). The forest plot (Fig. 2B) showed greater UT EMG activity during wobble board compared to stable surface (ES = 0.33, small; 95% CI [0.11–0.56]; *p* = 0.003; *I*^2^ = 0.0%; Egger’s test *p* = 0.347).

##### Stable surface compared to BOSU ball.

The comparison involved three studies (Tucker et al., 2010; Karagiannakis, Athanasopoulos & Mandalidis, 2018; De Araújo et al., 2020), involving 3 groups that completed push-ups on a stable surface (*n* = 53) and 3 groups that completed push-ups on BOSU ball (*n* = 53). The forest plot (Fig. 2C) showed similar UT EMG activity during BOSU ball compared to stable surface (ES = 0.30, small; 95% CI [−0.08–0.67]; *p* = 0.119; *I*^2^ = 0.0%; Egger’s test *p* = 0.123).

##### Stable surface compared to therapeutic ball.

The comparison involved three studies (Lehman, Gilas & Patel, 2008; Sandhu, Mahajan & Shenoy, 2008; Seo et al., 2013), involving 3 groups that completed push-ups on stable surface (*n* = 55) and 3 groups that completed push-ups on therapeutic ball (*n* = 55). The forest plot (Fig. 2D) showed greater UT EMG activity during therapeutic ball compared to stable surface (ES = 1.03, moderate; 95% CI [0.64–1.42]; *p* < 0.001; *I*^2^ = 0.0%; Egger’s test *p* = 0.753).

#### Serratus anterior

##### Stable surface compared to suspension equipment.

**Figure 3:**
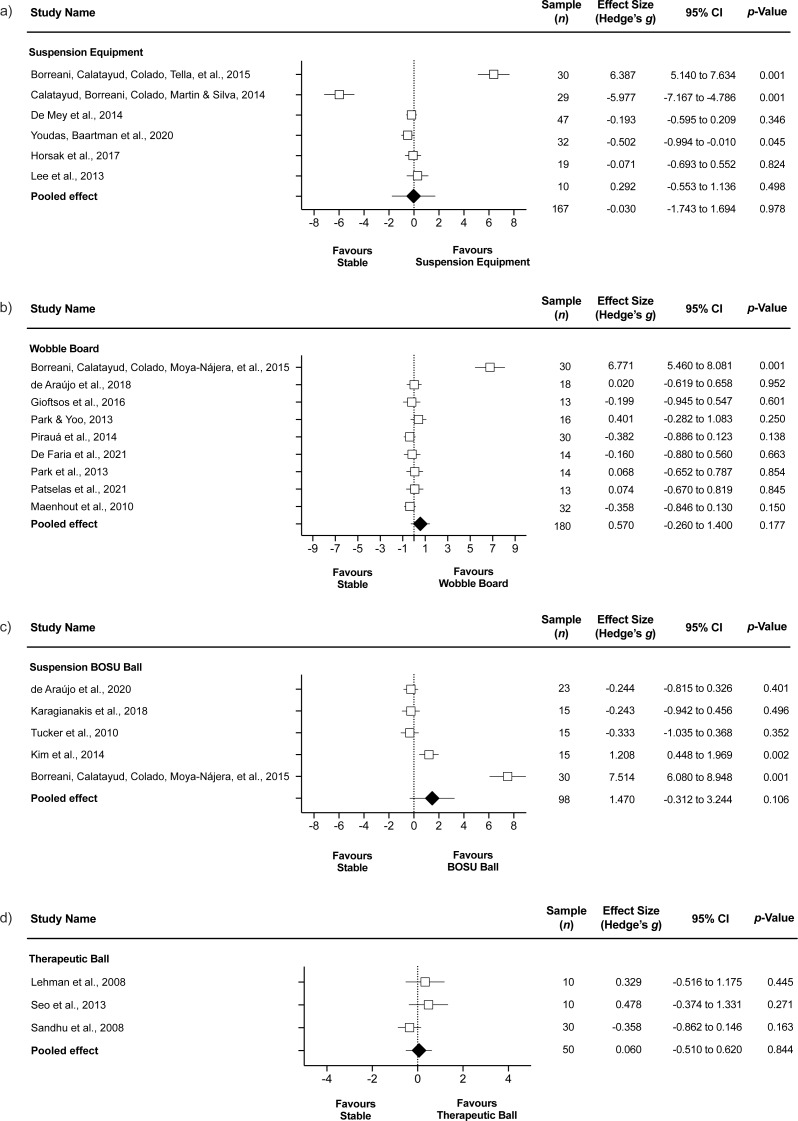
Serratus anterior EMG during push-ups performed on stable surface compared to (A) suspension equipment, (B) wobble board, (C) BOSU ball, and (D) therapeutic ball. Values shown are effect sizes (Hedges’s *g*) with 95% confidence intervals (CI).

The comparison involved six studies (Lee, Lee & Park, 2013; Calatayud et al., 2014b; De Mey et al., 2014; Borreani et al., 2015b; Horsak et al., 2017; Youdas et al., 2020a), involving six groups that completed push-ups on stable surface (*n* = 167) and 6 groups that completed push-ups on suspension equipment (*n* = 168). The forest plot (Fig. 3A) showed similar SA EMG activity during suspension equipment compared to stable surface (ES = −0.03, trivial; 95% CI [−1.74–1.69]; *p* = 0.978; *I*^2^ = 97.5%; Egger’s test *p* = 0.859). After a sensibility analysis with each study removed once from the meta-analysis to assess its impact on results heterogeneity, the results remained similar, with *I*^2^ values > 90%. Nonetheless, when a sensitivity analysis was conducted using the trim and fill method, the results indicated an ES = 1.54 and 95% CI [−0.54–3.62], favoring greater EMG for suspension equipment compared to stable surface.

##### Stable surface compared to wobble board.

The comparison involved nine studies (Maenhout et al., 2010; Park et al., 2013; Park & Yoo, 2013; Pirauá et al., 2014; Borreani et al., 2015a; Gioftsos et al., 2016; De Araújo et al., 2018; De Faria et al., 2021; Patselas et al., 2021), involving nine groups that completed push-ups on stable surface (*n* = 180) and 9 groups that completed push-ups on wobble board (*n* = 180). The forest plot (Fig. 3B) showed similar SA EMG activity during wobble board compared to stable surface (ES = 0.57, small; 95% CI [−0.26–1.40]; *p* = 0.177; *I*^2^ = 92.6%; Egger’s test *p* = 0.007). After a sensibility analysis with each study removed once from the meta-analysis to assess its impact on results heterogeneity, the results remained similar, with *I*^2^ values > 90%, except when a study was removed (Borreani et al., 2015a), as the *I*^2^ reached a value = 0.0%. Nonetheless, the *p* value remained non-significant (*p* = 0.276). The ES changed to −0.12 and the Egger’s test to *p* = 0.053, with values adjusted to ES = −0.25 and 95% CI [−0.46–−0.04].

##### Stable surface compared BOSU ball.

The comparison involved five studies (Tucker et al., 2010; Kim et al., 2014; Borreani et al., 2015a; Karagiannakis, Athanasopoulos & Mandalidis, 2018; De Araújo et al., 2020), involving five groups that completed push-ups on a stable surface (*n* = 98) and 5 groups that completed push-ups on a BOSU ball (*n* = 98). The forest plot (Fig. 3C) showed similar SA EMG activity during inverted Bosu compared to stable surface (ES = 1.47, large; 95% CI [−0.31–3.24]; *p* = 0.106; *I*^2^ = 96.4%; Egger’s test *p* = 0.015). After a sensibility analysis with each study removed once from the meta-analysis to assess its impact on results heterogeneity, the results remained similar, with *I*^2^ values > 90%, except when a study was removed (Borreani et al., 2015a), as the *I*^2^ reached a value = 74.4% (moderate). Nonetheless, the *p* value remained non-significant (*p* = 0.824). The ES changed to 0.08 and the Egger’s test *p* = 0.405, with values adjusted to ES = 0.21 and 95% CI [−0.39–0.81].

##### Stable surface compared to therapeutic ball.

The comparison involved three studies (Lehman, Gilas & Patel, 2008; Sandhu, Mahajan & Shenoy, 2008; Seo et al., 2013), involving 3 groups that completed push-ups on stable surface (*n* = 50) and three groups that completed push-ups on therapeutic ball (*n* = 50). The forest plot (Fig. 3D) showed similar SA EMG activity during therapeutic ball compared to stable surface (ES = 0.06, trivial; 95% CI [−0.51–0.62]; *p* = 0.844; *I*^2^ = 45.7%; Egger’s test *p* = 0.072).

### Certainty of evidence

The certainty of body of evidence was “Low” for an increased UT muscle activity in the wobble board compared to stable surface (*p* = 0.003), and therapeutic ball compared to stable surface condition (*p* < 0.001) (Table 3). In these meta-analyses performed, the initial level for descriptive studies was maintained, without downgrading for any criteria. On the other hand, “Very low” certainty was identified for the increase of UT muscle activity in suspension equipment compared to stable surface (*p* = 0.004), downgraded for inconsistency; and BOSU compared to stable surface condition (*p* = 0.0119), downgraded for imprecision. Regard to SA “very low” certainty of evidence indicate that there are not significative differences in the muscle activity on suspension equipment (*p* = 0.978) (Table 3), downgraded for inconsistency and imprecision; wobble board (*p* = 0.177), downgraded for imprecision and risk of publication bias; BOSU (*p* = 0.106), downgraded for imprecision risk of publication bias; and therapeutic ball (*p* = 0.844), downgraded for risk of bias of included studies (33% with high risk bias), and imprecision.

**Table 3:** Certainty of evidence for meta-analyzed outcomes.

**Outcome**	**Study design**	**ES (95% CI)**	***p*-value**	**N° studies** **(participants)**	**Risk of bias^a^**	**Inconsistency**	**Indirectness**	**Risk of publication bias^b^**	**Imprecision**	**Certainty of the evidence**
UT Stable *vs* Suspension	Descriptive	2,92 (0,92 to 4,92) Very large	0,004	6 (163/163)	No	Very large ↓	No	No	No	Very low
UT Stable *vs* Wobble board	Descriptive	0,33 (0,11 to 0,56) Small	0,003	8 (150/150)	No	Low	No	Yes ↓	No	Low
UT Stable *vs* BOSU	Descriptive	0,30 (−0,08 to 0,67) Small	0,011	3 (53/53)	No	Low	No	No	Yes ↓	Very low
UT Stable *vs* Therapeutic ball	Descriptive	1,03 (0,64 to 1,42) Moderate	0,001	3 (55/55)	No	Low	No	No	No	Low
SA Stable *vs* Suspension	Descriptive	-0,03 (−1.74 to 1.69) Trivial	0,978	6 (168/167)	No	Very large ↓	No	No	Yes ↓	Very low
SA Stable *vs* Wobble board	Descriptive	0,57 (−0.26 to 1.40) Small	0,177	9 (180/180)	No	Low	No	Yes ↓	Yes ↓	Very low
SA Stable *vs* BOSU	Descriptive	1,47 (−0.31 to 3.24) Large	0,106	5 (98/98)	No	Moderate	No	Yes ↓	Yes ↓	Very low
SA Stable *vs* Therapeutic ball	Descriptive	0,06 (−0.51 to 0.62) Trivial	0,844	3 (50/50)	Yes ↓ (33% high risk)	Moderate	No	No	Yes ↓	Very low

**Notes.**

UT upper trapezius SA serratus anterior ES effect size CI confidence interval ↓ Downgraded by one level

a Assessed with a standardized quality assessment form for observational studies (Siegfried et al., 2005) and it was adapted specifically for this study following recommendations of previous reports regarding risk of bias assessment of EMG studies (Ganderton & Pizzari, 2013; Edwards et al., 2017; Karabay, Emük & Özer Kaya, 2020; Cappato de Araújo et al., 2021).

b Assessed with Egger’s test (*p* < 0.05, risk of publication bias).

## Discussion

The systematic review of the evidence allowed to find 30 articles, in which EMG activity of SA and UT was analyzed during the execution of closed kinetic chain exercises, comparing a stable support surface with different types of unstable support surfaces. Unlike other studies, this meta-analysis is the first to compare different types of unstable support surfaces (*e.g.*, BOSU ball, therapeutic ball, and wobble board) with a stable surface (*e.g.*, floor), without combining them in a single pool of data.

The push-up variants (push-up, half push-up, and knee push-up) were the only exercises that fulfilled the criteria for the meta-analysis, showing in increasing order, a higher UT EMG activity when performed with wobble board (ES = 0.33), therapeutic ball (ES = 1.03) and suspension equipment (ES = 2.92) compared to a stable support surface, without observing any effect for the Bosu ball. According to GRADE recommendations (Guyatt et al., 2011; Zhang et al., 2019a; Zhang et al., 2019b), results must be interpreted with caution due to low quality of evidence of included studies, downgraded mostly by inconsistency (*i.e.*, *I*^2^ values > 75%) and imprecision (wide confidence intervals). Nonetheless, after sensitivity analyses, the results remained relatively consistent for all comparisons on UT myoelectrical activity. Based on the ES, these findings allow to establish a progression in the level of neuromuscular demand generated by different types of unstable support surfaces. This could be applied in the progressive prescription of scapular muscle training exercises and potentially in the rehabilitation of individuals with musculoskeletal dysfunctions of the shoulder complex.

From the point of view of the magnitude of the EMG activity, it was observed that the suspension equipment had an averaged increase of 9% of MVIC of the UT. This increase is higher than the observed in previous systematic reviews: Kang et al. (2019) showed a 2.85% MVIC increase and Cappato de Araújo et al. (2021) showed a 5.81% MVIC and 6.01% MVIC increase during push-up and half push-up, respectively. These differences could be explained because the articles included in the current meta-analyses were grouped according to the intrinsic characteristics of each instability device and its possible effects on neuromuscular recruitment.

In this way, the suspension equipment generates an asymmetric and higher shoulder complex instability, and additionally, a greater mobility of the proximal joints compared to distal joints, causing a greater displacement of the center of mass (Horsak et al., 2017), which seems to generate higher levels of EMG activation of the scapular and trunk stabilizer muscles compared with other types of unstable devices such as wobble board and therapeutic ball (Beach, Howarth & Callaghan, 2008; Anderson et al., 2013; Maeo et al., 2014; Calatayud et al., 2014a; Borreani et al., 2015a). The latter, on the contrary, have a bilateral symmetrical support, which would cause lesser demand on the scapular stabilizer muscles (Lehman et al., 2006). In addition, during the execution of a push-up with suspension equipment, the arm reaches around 90° of glenohumeral flexion accompanied by scapular elevation and upward rotation (De Mey et al., 2014; Borreani et al., 2015a). These scapular movements are caused by the action of UT (Kibler, Sciascia & Wilkes, 2012), which would justify the higher myoelectric activity observed in this muscle. On the other hand, UT had a higher EMG activity level on a therapeutic ball compared to a stable surface. In this sense, the therapeutic ball could generate a stimulus that would request a greater pushing force towards the ground to the distal muscles (*e.g.*, triceps brachii and biceps brachii) over the proximal muscles (*e.g.*, UT and SA) of the upper limb, due to the high deformation and mobility of the therapeutic ball under each hand (Lehman et al., 2006; Maeo et al., 2014; Bezerra et al., 2020), which could explain lower levels of neuromuscular activity compared to the suspension device.

On the other hand, no differences were identified in the SA EMG activity between different types of support surfaces. According to GRADE recommendations (Guyatt et al., 2011; Zhang et al., 2019a; Zhang et al., 2019b), results must be interpreted with caution due to imprecision, risk of bias, and inconsistency. In fact, after sensibility analyses using the trim and fill method, the results indicated greater SA EMG activity using suspension equipment compared to stable surface. In addition, after a study-by-study sensibility analyses, the removal of one study from the meta-analysis (Borreani et al., 2015a) indicated a small tendency toward greater EMG in stable surface compared to wobble board, although non-significant. This can be explained by an extremely low mean difference with respect to the rest of the studies, however, in the risk of bias analysis, no differences were observed in the internal validity items that would justify the exclusion of the study from the analysis.

These findings are similar to those observed by Kang et al. (2019), who did not observe effect of unstable surfaces on SA neuromuscular activity. Conversely, in his recent review Cappato de Araújo et al. (2021) showed a decrease in EMG activity of the SA while including unstable surfaces in one arm exercises, concluding that there was no advantage in the use of these surfaces in closed chain exercises. Nevertheless, these authors did not differentiate between different types of unstable surfaces in their analysis. In an effort to deepen this topic, previous studies analyzed the EMG activity of the SA using different unstable surfaces types (Herrington, Waterman & Smith, 2015; Horsak et al., 2017). The authors did not observe significant differences in SA activity between the support surfaces, concluding that these unstable devices would increase the neuromuscular activity of the glenohumeral and trunk stabilizers muscles to control the center of mass displacement rather than increase scapular muscle stabilizers EMG activity.

Rehabilitation programs establish that in early stages it is necessary to increase SA activation above UT to improve movement and stability of the scapulothoracic joint and reduce the risk of subacromial impingement (Ludewig & Cook, 2000; Kibler et al., 2008; Youdas et al., 2018). In this way, the support of the upper limb on unstable devices, such as suspension equipment or therapeutic ball, would be recommended to improve the activation levels of the UT once an adequate muscular balance has been achieved between the scapular rotator muscles. Additionally, other factors such as the location of the unstable device may lead to a more favorable activation of the SA compared to the UT. In this context, a greater myoelectric activity of SA has been reported when the unstable device is located below the lower limb during the execution of closed kinetic chain exercises (Byrne et al., 2014; Youdas et al., 2020a); however, future research is necessary to corroborate this.

### Limitations

The present investigation had limitations: (i) this report was based only on the EMG activity normalized by MVIC and did not consider the UT/SA ratio as a valid outcome measure for inter-subject comparisons. This measurement has been widely reported as the outcome that allows making comparisons between individuals and exercises, as well as this allows evaluating and analyzing the intra and inter-muscular balance (Ludewig et al., 2004; Berckmans et al., 2021); (ii) as reported by other reviews (Kang et al., 2019; Mendez-Rebolledo et al., 2021) most of the included studies did not present a priori a sample size or the statistical power of the analyzes, and they recruited participants healthy and asymptomatic, which make difficult to fully extrapolate the results of this research to populations with shoulder musculoskeletal dysfunctions; (iii) 93,3% the included studies showed a moderate methodological quality, mainly due internal validity aspects; and (iv) the present investigation was not able to carry out a meta-analysis for the push-up plus exercise, considering that add scapular protraction may increment the myoelectrical activity of the SA, involved directly in this movement. Finally, and according to GRADE, the certainty of evidence ranged from “very low” to “low” for the outcomes, harming the confidence in the presented estimates.

### Perspectives

The research raises the possibility of prescribing exercises with different degrees of neuromuscular demands for UT in healthy subjects, according to the type of unstable support surface used, which was not observed for SA. This progressivity of the UT occurs when different unstable support surfaces are not combined in a single data set for the meta-analysis. However, it was not possible to make comparisons between different unstable support surfaces (*e.g.*, suspension equipment v/s BOSU; suspension equipment v/s therapeutic ball; BOSU v/s therapeutic ball) due to the lack of studies that performed this type of analysis. For these reasons, we recommend that future research try to determine possible differences in the myoelectric activity of UT and SA between unstable support surfaces in healthy subjects and with musculoskeletal disorders of the shoulder, rather than just comparing a stable surface with an unstable one.

## Conclusions

Different unstable support surfaces induce an increase in the myoelectric activity of the UT during a push-up performance, suggesting based on ES, a progressive neuromuscular demand, specifically higher EMG activity when using wobble board, therapeutic ball, and suspension equipment. Conversely, no differences were observed in SA, which could mean that performing a push up on unstable surfaces does not affect SA muscle activity level. However, these findings must be interpreted with caution due to reduced certainty of evidence for most outcomes. Nonetheless, current findings could be applied in the prescription of scapular muscle training exercises in healthy individuals and potentially in the rehabilitation of individuals with musculoskeletal dysfunctions of the shoulder complex, allowing to properly select the appropriate unstable device for a given stage of rehabilitation or training program.

## Supplemental Information

10.7717/peerj.13589/supp-1 Appendix S1 The combination of key words and Boolean operators used to retrieve the studies

10.7717/peerj.13589/supp-2 Supplemental Information 2 PRISMA 2020 Abstract Checklist

10.7717/peerj.13589/supp-3 Supplemental Information 3 PRISMA 2020 Main Checklist

10.7717/peerj.13589/supp-4 Data S1 Data used for meta-analysis EMG mean ± standard deviation (SD) of scapular muscles during push-ups on different surfaces. The data were standardized using post score SD.

10.7717/peerj.13589/supp-5 Supplemental Information 5 Rationale for conducting the systematic review/meta-analysis and contribution the research makes to knowledge in light of previously published related reports

## Additional Information and Declarations

### Data Availability

The following information was supplied regarding data availability: The raw data is available in the Supplemental File.
