# Supplementary material for: Electromyographic analysis of the serratus anterior and upper trapezius in closed kinetic chain exercises performed on different unstable support surfaces: a systematic review and meta-analysis
Source: PeerJ. 2022 Jun 30;10:e13589. doi: 10.7717/peerj.13589 (PMC9250763; doi:10.7717/peerj.13589)
Supplement: Appendix S1 [file peerj-10-13589-s001.docx]

**Appendix A.** The following combination of key words and Boolean operators were used to retrieve the studies:

*Pubmed Central:*

Filters Pubmed: humans, english, since 01-01-1995 to 09-30-2021.

1. scapular muscles (Text Word)
2. upper trapezius (Text Word)
3. serratus anterior (Text Word)
4. 1 OR 2 OR 3
5. muscle activation (Text Word)
6. muscle activity (Text word)
7. recruitment (Text Word)
8. 5 OR 6 OR 7
9. electromyography (MeSH Terms)
10. emg (Text Word)
11. 9 OR 10
12. exercise (MeSH Terms)
13. training (MeSH Terms)
14. 13 OR 14
15. Instability
16. Unstable surface
17. Unstable support
18. Labile surface
19. 15 OR 16 OR 17 OR 18
20. 4 AND 8 AND 11 AND 14 AND 19

*SPORTDiscus*

Limit: English, human, article, since 01-01-1995 to 09-30-2021.

1. scapular muscles OR serratus anterior OR upper trapezius
2. muscle activation OR muscle activity OR recruitment
3. electromyography OR emg
4. exercise OR training
5. Instability OR unstable surface OR unstable support OR labile surface
6. 1 AND 2 AND 3 AND 4 AND 5

*ScienceDirect*

Filters: Nursing and health science, since 1995 to 2021, and research articles

1. Scapular muscles
2. Upper trapezius
3. Serratus anterior
4. 1 OR 2 OR 3
5. Muscle activation
6. Electromyography
7. Exercise
8. Unstable Surface
9. Instability
10. 8 OR 9
11. 4 AND 5 AND 6 AND 7 AND 10
