# Supplementary material for: Electromyographic analysis of the serratus anterior and upper trapezius in closed kinetic chain exercises performed on different unstable support surfaces: a systematic review and meta-analysis
Source: PeerJ. 2022 Jun 30;10:e13589. doi: 10.7717/peerj.13589 (PMC9250763; doi:10.7717/peerj.13589)
Supplement: Supplemental Information 3 [file peerj-10-13589-s003.pdf]

PRISMA 2020 Main Checklist

Topic	No.	Item	Location where item is reported
**TITLE**			
**Title**	1	Identify the report as a systematic review.	Line 1-4
**ABSTRACT**			
**Abstract**	2	See the PRISMA 2020 for Abstracts checklist	Line 27-57
**INTRODUCTION**			
**Rationale**	3	Describe the rationale for the review in the context of existing knowledge.	Line 83-109
**Objectives**	4	Provide an explicit statement of the objective(s) or question(s) the review addresses.	Line 109-113
**METHODS**			
**Eligibility criteria**	5	Specify the inclusion and exclusion criteria for the review and how studies were grouped for the syntheses.	Line 125-143
**Information sources**	6	Specify all databases, registers, websites, organisations, reference lists and other sources searched or consulted to identify studies. Specify the date when each source was last searched or consulted.	Line 146-149
**Search strategy**	7	Present the full search strategies for all databases, registers and websites, including any filters and limits used.	Line 149-151 and Appendix_A
**Selection process**	8	Specify the methods used to decide whether a study met the inclusion criteria of the review, including how many reviewers screened each record and each report retrieved, whether they worked independently, and if applicable, details of automation tools used in the process.	Line 154-157
**Data collection process**	9	Specify the methods used to collect data from reports, including how many reviewers collected data from each report, whether they worked independently, any processes for obtaining or confirming data from study investigators, and if applicable, details of automation tools used in the process.	Line 159-167

Topic	No.	Item	Location where item is reported
**Data items**	10a	List and define all outcomes for which data were sought. Specify whether all results that were compatible with each outcome domain in each study were sought (e.g. for all measures, time points, analyses), and if not, the methods used to decide which results to collect.	Line 170-172
	10b	List and define all other variables for which data were sought (e.g. participant and intervention characteristics, funding sources). Describe any assumptions made about any missing or unclear information.	Line 172-175
**Study risk of bias assessment**	11	Specify the methods used to assess risk of bias in the included studies, including details of the tool(s) used, how many reviewers assessed each study and whether they worked independently, and if applicable, details of automation tools used in the process.	Line 177-189
**Effect measures**	12	Specify for each outcome the effect measure(s) (e.g. risk ratio, mean difference) used in the synthesis or presentation of results.	Line 210-215
**Synthesis methods**	13a	Describe the processes used to decide which studies were eligible for each synthesis (e.g. tabulating the study intervention characteristics and comparing against the planned groups for each synthesis (item 5)).	Line 172-175 and Table 1
	13b	Describe any methods required to prepare the data for presentation or synthesis, such as handling of missing summary statistics, or data conversions.	Line 164-167 and line 210-213
	13c	Describe any methods used to tabulate or visually display results of individual studies and syntheses.	Table 1
	13d	Describe any methods used to synthesize results and provide a rationale for the choice(s). If meta-analysis was performed, describe the model(s), method(s) to identify the presence and extent of statistical heterogeneity, and software package(s) used.	Line 215-229
	13e	Describe any methods used to explore possible causes of heterogeneity among study results (e.g. subgroup analysis, meta-regression).	Line 220-224
	13f	Describe any sensitivity analyses conducted to assess robustness of the synthesized results.	Line 222-227
**Reporting bias assessment**	14	Describe any methods used to assess risk of bias due to missing results in a synthesis (arising from reporting biases).	Line 225-227

Topic	No.	Item	Location where item is reported
**Certainty assessment**	15	Describe any methods used to assess certainty (or confidence) in the body of evidence for an outcome.	Line 191-207
**RESULTS**			
**Study selection**	16a	Describe the results of the search and selection process, from the number of records identified in the search to the number of studies included in the review, ideally using a flow diagram.	Line 232-235 and Figure 1
	16b	Cite studies that might appear to meet the inclusion criteria, but which were excluded, and explain why they were excluded.	Line 235-237
**Study characteristics**	17	Cite each included study and present its characteristics.	Line 241-251 and Table 1
**Risk of bias in studies**	18	Present assessments of risk of bias for each included study.	Line 255-283 and Table 2
**Results of individual studies**	19	For all outcomes, present, for each study: (a) summary statistics for each group (where appropriate) and (b) an effect estimate and its precision (e.g. confidence/credible interval), ideally using structured tables or plots.	Line 287-367 and Figure 2 and 3
**Results of syntheses**	20a	For each synthesis, briefly summarise the characteristics and risk of bias among contributing studies.	Line 255-283
	20b	Present results of all statistical syntheses conducted. If meta-analysis was done, present for each the summary estimate and its precision (e.g. confidence/credible interval) and measures of statistical heterogeneity. If comparing groups, describe the direction of the effect.	Line 287-367 and Figure 2 and 3
	20c	Present results of all investigations of possible causes of heterogeneity among study results.	Line 371-384<br>Table 3
	20d	Present results of all sensitivity analyses conducted to assess the robustness of the synthesized results.	Line 287-367
**Reporting biases**	21	Present assessments of risk of bias due to missing results (arising from reporting biases) for each synthesis assessed.	Line 371-384<br>Table 3
**Certainty of evidence**	22	Present assessments of certainty (or confidence) in the body of evidence for each outcome assessed.	Line 371-384<br>Table 3
**DISCUSSION**			
**Discussion**	23a	Provide a general interpretation of the results in the context of other evidence.	Line 389-468

Topic	No.	Item	Location where item is reported
	23b	Discuss any limitations of the evidence included in the review.	Line 399-407<br>Line 336-445
	23c	Discuss any limitations of the review processes used.	Line 470-485
	23d	Discuss implications of the results for practice, policy, and future research.	Line 458-468<br>Line 487-498<br>Line 500-510
**OTHER INFORMATION**			
**Registration and protocol**	24a	Provide registration information for the review, including register name and registration number, or state that the review was not registered.	Line 56
	24b	Indicate where the review protocol can be accessed, or state that a protocol was not prepared.	Line 56
	24c	Describe and explain any amendments to information provided at registration or in the protocol.	Not apply
**Support**	25	Describe sources of financial or non-financial support for the review, and the role of the funders or sponsors in the review.	Line 56-57
**Competing interests**	26	Declare any competing interests of review authors.	Declared in PeerJ submit process
**Availability of data, code and other materials**	27	Report which of the following are publicly available and where they can be found: template data collection forms; data extracted from included studies; data used for all analyses; analytic code; any other materials used in the review.	Data used for all analyses: declared in PeerJ submit process
