# Supplementary material for: Electromyographic analysis of the serratus anterior and upper trapezius in closed kinetic chain exercises performed on different unstable support surfaces: a systematic review and meta-analysis
Source: PeerJ. 2022 Jun 30;10:e13589. doi: 10.7717/peerj.13589 (PMC9250763; doi:10.7717/peerj.13589)
Supplement: Supplemental Information 5 [file peerj-10-13589-s005.docx]

**Rationale for conducting the systematic review/meta-analysis and contribution the research makes to knowledge in light of previously published related reports:**

As a mean of progression in muscle strengthening and rehabilitation programs, unstable support surfaces have been incorporated into the push-up variants. Compared to stable surface, increased serratus anterior (SA) and upper trapezius (UT) EMG during push-ups has been reported when performed on unstable surfaces such as both-sides-up (BOSU) ball (Borreani et al., 2015; Tucker et al., 2010), wobble board (Biscarini et al., 2019; Park & Yoo, 2011), therapeutic ball (Seo et al., 2013), and suspension equipment (De Mey et al., 2014; Jeong et al., 2014). However, contradictory results have been reported, observing a significant decrease or no difference in the EMG of the SA when comparing different types of unstable surfaces against stable surface (Gioftsos et al., 2016; Horsak et al., 2017; Pirauá et al., 2014). **Previous systematic reviews** showed that push-up exercises performed on unstable support surfaces increased UT EMG compared to a stable surface, without an effect in the SA muscle (Cappato de Araújo et al., 2021; Kang et al., 2019). **However, the comparative analyzes observed in those reports have grouped in a single data pool different types of unstable support surfaces –BOSU, therapeutic ball, suspension equipment, among others– without considering the possible and potential differences in neuromuscular demand induced by the individual analysis of each unstable support surface** (Mendez-Rebolledo et al., 2021)**. In this context, to our knowledge, there is no** **quantitative analysis of the evidence that groups the data according to these differences.**

For these reasons, it is necessary to carry out a systematic search of the literature that considers inclusion and exclusion criteria related to the design and execution of the main exercises in closed kinetic chain of the upper limb –push-up, push-up plus, plank, scap protraction– and at the same time distinguish between different unstable support surfaces, without combining them in a single pool of data. The findings of this research could be applied in the prescription of scapular muscle training exercises in healthy individuals and potentially in the rehabilitation of individuals with musculoskeletal dysfunctions of the shoulder complex.

Biscarini, A., Contemori, S., & Grolla, G. (2019). Activation of Scapular and Lumbopelvic Muscles During Core Exercises Executed on a Whole-Body Wobble Board. *Journal of Sport Rehabilitation*, *28*(6), 623-634. https://doi.org/10.1123/jsr.2018-0089

Borreani, S., Calatayud, J., Colado, J. C., Moya-Nájera, D., Triplett, N. T., & Martin, F. (2015). Muscle activation during push-ups performed under stable and unstable conditions. *Journal of Exercise Science & Fitness*, *13*(2), 94-98. https://doi.org/10.1016/j.jesf.2015.07.002

Cappato de Araújo, R., Andrade da Silva, H., Pereira dos Passos, M. H., Alves de Oliveira, V. M., & Rodarti Pitangui, A. C. (2021). Use of unstable exercises in periscapular muscle activity: A systematic review and meta-analysis of electromyographic studies. *Journal of Bodywork and Movement Therapies*, *26*, 318-328. https://doi.org/10.1016/j.jbmt.2020.12.010

De Mey, K., Danneels, L., Cagnie, B., Borms, D., T’Jonck, Z., Van Damme, E., & Cools, A. M. (2014). Shoulder Muscle Activation Levels During Four Closed Kinetic Chain Exercises With and Without Redcord Slings. *Journal of Strength and Conditioning Research*, *28*(6), 1626-1635. https://doi.org/10.1519/JSC.0000000000000292

Gioftsos, G., Arvanitidis, M., Tsimouris, D., Kanellopoulos, A., Paras, G., Trigkas, P., & Sakellari, V. (2016). EMG activity of the serratus anterior and trapezius muscles during the different phases of the push-up plus exercise on different support surfaces and different hand positions. *Journal of Physical Therapy Science*, *28*(7), 2114-2118. https://doi.org/10.1589/jpts.28.2114

Horsak, B., Kiener, M., Pötzelsberger, A., & Siragy, T. (2017). Serratus anterior and trapezius muscle activity during knee push-up plus and knee-plus exercises performed on a stable, an unstable surface and during sling-suspension. *Physical Therapy in Sport*, *23*, 86-92. https://doi.org/10.1016/j.ptsp.2016.08.003

Jeong, S. Y., Chung, S. H., & Shim, J. H. (2014). Comparison of Upper Trapezius, Anterior Deltoid, and Serratus Anterior Muscle Activity during Push-up plus Exercise on Slings and a Stable Surface. *Journal of Physical Therapy Science*, *26*(6), 937-939. https://doi.org/10.1589/jpts.26.937

Kang, F.-J., Ou, H.-L., Lin, K.-Y., & Lin, J.-J. (2019). Serratus Anterior and Upper Trapezius Electromyographic Analysis of the Push-Up Plus Exercise: A Systematic Review and Meta-Analysis. *Journal of Athletic Training*, *54*(11), 1156-1164. https://doi.org/10.4085/1062-6050-237-18

Mendez-Rebolledo, G., Morales-Verdugo, J., Orozco-Chavez, I., Habechian, F. A. P., Padilla, E. L., & de la Rosa, F. J. B. (2021). Optimal activation ratio of the scapular muscles in closed kinetic chain shoulder exercises: A systematic review. *Journal of Back and Musculoskeletal Rehabilitation*, *34*(1), 3-16. https://doi.org/10.3233/BMR-191771

Park, S., & Yoo, W. (2011). Differential activation of parts of the serratus anterior muscle during push-up variations on stable and unstable bases of support. *Journal of Electromyography and Kinesiology*, *21*(5), 861-867. https://doi.org/10.1016/j.jelekin.2011.07.001

Pirauá, A. L. T., Pitangui, A. C. R., Silva, J. P., dos Passos, M. H. P., de Oliveira, V. M. A., Batista, L. da S. P., & de Araújo, R. C. (2014). Electromyographic analysis of the serratus anterior and trapezius muscles during push-ups on stable and unstable bases in subjects with scapular dyskinesis. *Journal of Electromyography and Kinesiology*, *24*(5), 675-681. https://doi.org/10.1016/j.jelekin.2014.05.009

Seo, S.-H., Jeon, I.-H., Cho, Y.-H., Lee, H.-G., Hwang, Y.-T., & Jang, J.-H. (2013). Surface EMG during the Push-up plus Exercise on a Stable Support or Swiss Ball: Scapular Stabilizer Muscle Exercise. *Journal of Physical Therapy Science*, *25*(7), 833-837. https://doi.org/10.1589/jpts.25.833

Tucker, W. S., Armstrong, C. W., Gribble, P. A., Timmons, M. K., & Yeasting, R. A. (2010). Scapular Muscle Activity in Overhead Athletes With Symptoms of Secondary Shoulder Impingement During Closed Chain Exercises. *Archives of Physical Medicine and Rehabilitation*, *91*(4), 550-556. https://doi.org/10.1016/j.apmr.2009.12.021
